# Core and modifiable components of academic detailing: demonstration of implementation strategy development, tailoring, and documentation process

**DOI:** 10.3389/frhs.2025.1521504

**Published:** 2025-06-03

**Authors:** Ariel M. Domlyn, Gwendolyn Hooks, Michelle Freitag, Lacey Evans, Madison Stewart, Laura Damschroder, Jeremy B. Sussman

**Affiliations:** ^1^ Center for Clinical Management Research, VA Ann Arbor Healthcare System, Ann Arbor, MI, United States; ^2^ Division of General Medicine, University of Michigan, Ann Arbor, MI, United States

**Keywords:** academic detailing, implementation strategies, implementation science, tailoring strategy, QUERI, FRAME-IS

## Abstract

**Background:**

Academic Detailing (AD) is an educational outreach strategy that has shown positive effects on clinical practice, but its implementation varies widely across programs, necessitating consistent definitions of its essential components. The lack of standardized guidance for tailoring AD and other multi-component implementation strategies presents challenges in program development and effectiveness evaluation. To address this, we applied FRAME-IS (Framework for Reporting Adaptations and Modifications to Evidence-based Implementation Strategies) to specify AD's core components and demonstrate a repeatable program development process. By showcasing a multi-project, multi-site AD program, we aim to provide guidance for others in developing and tailoring AD programs, ultimately enabling more rigorous evaluations of AD's effectiveness.

**Methods:**

Literature and training materials were reviewed to develop a list of common AD components, then organized according to a FRAME-IS template. Coders applied directed content analysis to materials from the MIDAS AD program, a multi-site implementation center using AD in four projects across the Veterans Health Administration. Tailoring and development of the AD program was coded according to FRAME-IS modules and ERIC strategy taxonomy.

**Results:**

18 common AD components were identified. These components were retained but six were tailored and an additional seven were added across the MIDAS projects. The rationale for tailoring and additions was mostly to increase appropriateness, acceptability, adoption, and reach of AD. To assist in future tailoring of AD programs, we developed a list of generalizable guiding questions and an AD program documentation and tailoring template.

**Conclusions:**

AD is a robust strategy, but empirical study of the core and modifiable components is constrained by variable definitions of the components. This is the first attempt at developing documentation and tailoring guidelines for AD programs using the nomenclature of implementation science. We further suggest which components may be core and which may be modifiable. Our effort to specify AD components using the FRAME-IS method provides an example for other AD programs, contributing to the future use and study of AD as an implementation strategy and paving the way for more rigorous analysis of which modifications affect outcomes.

**Trial registration**: ClinicalTrials.gov: NCT05065502.

## Introduction

For complex behavioral interventions, tailoring implementation strategies to address context is the rule, not an exception (1, 2). But tools to aid effective, conceptually sound, and efficient modification are only recently being developed (3, 4). Before tailoring or modification begins, consistent definitions of strategy components are essential. One strategy with a history of modifying components is Academic Detailing (AD). In use since the 1980s, AD is a form of educational outreach in which trained staff (“detailers”) have one-on-one conversations with healthcare providers to improve those providers' adoption of specific evidence-based practices (EBPs) (5, 6). This strategy has been widely and successfully used within the pharmaceutical industry (5–10). Yet experts in AD acknowledge a wide range of conceptualizations regarding which components are necessary for a program to be considered “Academic Detailing.” Individual AD programs vary in design, approach, and structure, including basic choices such as whether detailers need professional credentialing vs. project-specific training and whether educational interactions must be one-on-one vs. a group format (5–8, 11, 12).

Not only do the many structures of AD programs make program development challenging, but there is also no guidance about which design modifications to choose to best meet the needs of a specific AD program. This problem is common in multi-component implementation strategies, which are rarely fully described in the literature or are presented as unbreakable multi-component packages (3, 13), creating a challenge for both the study of their effect and tailoring efforts. Further, it is often unclear when, how, and why to tailor strategies (14). This concern has led to attempts to identify, track, and more clearly document modifications made to implementation strategies (4, 15). The Framework for Reporting Adaptations and Modifications to Evidence-based Implementation Strategies (FRAME-IS) provides a modular template with detailed guidance for specifying components and adaptations (4, 16–18). It is highly modular which allows thorough specification of both core and modifiable components of a strategy that has a diversity of conceptualizations. Application of FRAME-IS to AD allows us to create an AD framework for rational modification of AD programs.

This paper aims to describe possible modifications in AD when using as an implementation strategy and to provide guidance for developing and tailoring an AD program. Specifically, we aim to: (1) Specify AD core components with FRAME-IS nomenclature; (2) Demonstrate a repeatable program development process using FRAME-IS to describe the “how” and “why” of tailoring for developing one AD program used in four implementation projects; and (3) Provide guidance for developing an AD program. The context was our development of a multi-project, multi-site AD program within the United States Veterans Health Administration (VHA), and we present examples of modifications we considered and ultimately chose. This work is novel both in its use of FRAME-IS as a generalizable template for AD program development and tailoring, and in defining the core vs. modifiable components of AD that provides a guide for others to follow. While it is beyond our current scope to assess the effect of program modifications on AD outcomes, we lay the groundwork to enable rigorous evaluations of AD effectiveness.

## Materials and methods

### Creation of AD tailoring documentation system

FRAME-IS provides a template for systematically tracking modifications to an implementation strategy according to discrete modules (4). The modules report the: (1) “Description” of the EBP, implementation strategy, and brief modification description. (2) “What” (type) of strategy modification made according to four categories: content of the strategy itself or its delivery, evaluation for the way the strategy is evaluated, training in terms of the manner of implementer training, and context of how the strategy is delivered, specifically changes to format of delivery, setting of delivery, personnel delivering the strategy, or population targeted for delivery. (3) “Nature” of modification, which refers to the extent and intensity of the modification with pre-specified codes such as tailoring; adding elements; removing elements; lengthening, substituting, spreading, or integrating another strategy into the implementation strategy in primary use; loosening structure; and others. (4) “Rationale” behind the modification. This defines both the goal and level of modification. (4a) Goal codes are sorted by implementation outcomes such as intending to improve reach, effectiveness, adoption, acceptability, appropriateness, feasibility, fidelity, cost, sustainability, or equity. (4b) Level codes dictate identifying the socioecological levels influencing the decision to modify for sociopolitical, organizational, implementer, provider, or patient reasons. (5) “When” (timing) of tailoring the strategy components (pre-implementation, implementation, scale-up, or maintenance) and whether the modification was planned/proactive, planned/reactive, or unplanned/reactive. (6) “Who” defined or is involved in the decision to modify the strategy: political leaders, program leaders, funder, implementer, researcher, providers, community members, or patients.

Consistent with other implementation scientists (14, 15, 19), we use “tailoring” to reference altering strategies to fit context and “modification” as a generic term for change. There is debate but little consensus regarding the scope and definitions of the terms “adaptation,” “tailoring,” and “modification.” Notably, some researchers may classify AD tailoring as an “adaptive implementation strategy” (2, 20).

### Identifying common components of AD

To understand and guide modifications, we agreed on a set of components common to AD. Due to the vast range of AD conceptualizations in the literature (5–8, 10–12, 21, 22), the decision was made to identify common components from publicly available implementation and training materials (23–25). Priority was given to AD components named by the National Resource Center for Academic Detailing (NaRCAD) and the VHA Pharmacy Benefits Management's Academic Detailing Services (ADS), as both are national leaders in AD development and practice. One project investigator (AMD) cross-walked these training materials to identify 18 components that tend to exist in AD programs both within and beyond VHA. Components were grouped by the FRAME-IS module 2 (“What”) categories (content, evaluation, training, and context) to create an initial matrix of the AD common components. The 18 components were further typed according to the Expert Recommendations for Implementing Change (ERIC) taxonomy of implementation strategies (26) using a coding specification tool (27). ERIC is a sorted list of implementation strategies derived from expert consensus and concept mapping. The phrasing used in ERIC has become common nomenclature in implementation science. ERIC terms are widely used to ensure implementation tools are generalizable across contexts and innovations, and to assist future meta-analyses of implementation strategy effectiveness. Coding strategy components to ERIC is not prescribed by FRAME-IS developers but was conducted here to standardize strategy terms. By standardizing AD component definitions to a general taxonomy, this will enable future AD analyses and ensure that AD modifications are consistently defined.

### Maintaining implementation through dynamic adaptations (MIDAS) AD program development

An overarching AD program was developed to support AD projects for three hybrid III cluster randomized implementation trials (28) and a non-randomized intervention project as part of the Maintaining Implementation through Dynamic Adaptations (MIDAS) Quality Enhancement Research Initiative (QUERI) center. Within each of these projects, AD was used as a strategy to support a specific quality improvement program (29) within participating healthcare sites across VHA. The goals for the four projects were: to reduce inappropriate polypharmacy among older adults, improve safe use of Direct Oral Anticoagulants (DOACs), promote Cognitive Behavioral Therapy for Insomnia (CBT-I) as a first-line treatment over medications, and increase referrals to a telehealth suicide prevention program. The MIDAS AD program deployed in 23 sites across VHA from 2021–2024 to support the four projects.

The MIDAS team developed an AD program to support these projects. The AD program needed to meet four key criteria and work within immutable constraints. First, the program must be able to support the broad range of project topics. Second, we were not able to support separate detailers with content expertise for each of the four project topics. Third, national policies prevented us from employing VHA providers or funding VHA ADS to staff the projects (6, 9, 10, 30). Fourth, rigorous evaluation required consistency across projects, clear definition of AD strategies used, clear descriptions of adaptations, and clearly defined outcomes. MIDAS AD program development was informed by a comprehensive review of existing AD program materials, particularly from the VHA ADS (23), NaRCAD (24), and existing research publications (5–7, 11, 12, 21) and from our experience with quality improvement and implementation science interventions. Based on these sources, we created a master protocol specifying project content, evaluation design, and detailer training tailored to MIDAS's needs (Supplementary Material A). Among these materials are specifications of evaluation tools including a fidelity assessment using self-ratings and peer review (Supplementary Material B), a detailer script (Supplementary Material C), and a participant satisfaction survey (Supplementary Material D).

### Classification of MIDAS AD components

To classify MIDAS modifications, we followed five steps for rigorous document analysis (31–34). First, two trained detailers (GH, MF) independently defined the MIDAS AD program components by conducting a MIDAS document review using modified directed content analysis (31, 32). We reviewed materials from across the development and implementation of the four projects including protocol drafts, training materials, fidelity assessment entries (such as post-detailing session notes and ratings), and peer reviewer notes. Second, we classified components according to the FRAME-IS modules. One investigator (AMD) applied predetermined FRAME-IS module codes (4) to component descriptions for MIDAS AD, then re-coded components based on ERIC labels and definitions. Labels were applied based on identifying the best fit between detailed MIDAS AD component descriptions and ERIC labels provided by the developers (26, 27), with an emphasis on the function of the component. As needed, the investigator consulted with an ERIC developer (LD) for accuracy. Third, two collaborators (LE, MS) iteratively reviewed the MIDAS AD component descriptions, categorizations, and codes as a form of validity check via peer debriefing (33). Fourth, for reliability checks (34), content and codes were reviewed and revised in five reflexivity discussions held over the course of three months with the investigative team (GH, MF, AMD, LE, MS). Finally, the final reporting structure was reviewed and approved by two project leaders (JBS, LD) as representative of the MIDAS AD program.

## Results

### Academic detailing documentation system

The AD documentation system used FRAME-IS codes for each module (Table 1). These definitions were extracted from the method developers (4) and worded to be AD-specific. The AD tailoring definitions are the codes within each module we suggest for use when documenting AD program customization to context, as in MIDAS (below). Supplementary Material E provides a blank AD FRAME-IS template for other programs to describe their AD components and modifications.

**Table 1:** Academic detailing (AD) documentation system based on FRAME-IS.

FRAME-IS Module	AD Modification Definition
1. Description of the AD program	Evidence-Based Practice (EBP): The innovation or campaign in which AD is used.
	Strategy: AD, an educational outreach strategy.
2. What is tailored	Content: Changes to strategy content of AD itself, or that impact delivery of detailing.
	Evaluation: Changes to the way AD is evaluated.
	Training: Changes to how AD personnel are trained or prepared.
	Context: Changes to AD delivery. Includes changes to format, setting, personnel, and population.
3. Nature of tailoring	Adding, Removing, Substitution, Tweaking, Refining, Integrating another strategy, etc.
4. Rationale and Goal of adaptation	Reach: Number or representativeness of the population detailed.
	Adoption: Intended population agreeing to be detailed.
	Feasibility: Extent that AD can be carried out within a setting.
	Acceptability: Perception of recipient satisfaction with detailing.
	Appropriateness: Perception of how well detailing fits within the setting.
	Fidelity: Degree that detailing was conducted with rigor and consistency.
	Sustainability: Extent that AD may continue to be used within the setting.
	Cost: Cost impact of AD, fiscal and temporal.
5. Level of adaptation	Practitioner: Those being detailed about the EBPs.
	Implementer: Academic detailer and support team.
	Organizational level: Clinic, hospital, or regional healthcare setting.
	Sociopolitical level: National mandates or norms.
6. When the adaptation was initiated and whether it was planned	Pre-implementation, Implementation, Scale-up, Maintenance, Sustainment
	Reactivity
	- Planned/Proactive: Intentionally by anticipating setting needs. - Planned/Reactive: Intentionally in response to emergent setting needs. - Unplanned/Reactive: Unintentionally in response to emergent setting needs.
7. Who was involved in the decision to modify	Healthcare system leaders, AD program leaders, AD funder, AD practitioner, researcher, AD recipient (practitioners), or patients.

### Common components of AD

We identified common core components and subcomponents of AD programs structured by the FRAME-IS “what” module (Table 2). Content comprises three core components: campaign development, campaign initiation and recruitment, and in-session delivery. Each component has subcomponents; for example, developing key messages, meeting with clinical staff, and delivering a presentation about the topic. The only common component for AD evaluation is collecting outreach process data to document which providers were educated, the duration and timing of those educational outreach sessions, and the content of the session such as barriers brought up by the provider (for example, a necessary medication is not in their order set) and whether the detailer secured the provider's verbal commitment to change their prescribing behavior to be more consistent with the innovation. The three common components for training dictate having detailers attend a basic skills workshop, meeting with detailer colleagues or consultants for consultation, and practicing via role play. Finally, four common components of context show that AD outreach visits are usually delivered one-on-one, in-person, to front-line staff, and by someone with a background in a health-related field.

**Table 2:** Academic detailing (AD) common components per FRAME-IS “what” code and ERIC naming convention.

FRAME-IS “What” (Module 2): Categories	Common AD Component	Common AD Subcomponent	ERIC Strategy Label
**Content** The implementation strategy itself, or that impact how aspects of the implementation strategy are delivered	Campaign Development	Use behavior change theories to inform outreach strategy	N/A
		Set goal of improving adherence to evidence-based practice (EBP)	Develop a formal implementation blueprint
		Develop key messages based on evidence for topic	Tailor strategies
		Develop EBP materials (e.g., practitioner guide, quick reference guide, patient materials)	Develop educational materials
	Campaign Initiation and Recruitment	Create templates for recruitment communication (e.g., email scripts, call scripts)	N/A
		Meet with clinical staff	Conduct educational outreach visit
	In-session Delivery	Use NaRCAD process model: Intro, needs assessment, key messages, handling objections, summary, closing	Make training dynamic
		Deliver a presentation about research on the topic	Conduct educational outreach visit
		Use motivational language to overcome barriers	Make training dynamic
		Provide clinician with EBP materials (e.g., brochures, 1-pager, pocket cards)	Distribute educational materials
**Evaluation** The way that the implementation strategy is evaluated	Collect outreach data (clinician demographics, method of outreach, length of visit, key messages covered, barriers, commitment to behavior change)		Develop and implement tools for quality monitoring
**Training** The ways that implementers are trained	Train the detailer in basic AD skills.		Train facilitator
	Recurring meetings to discuss cases		Provide ongoing consultation
	Role plays/opportunities to practice		Make training dynamic
**Context** The way the overall implementation strategy is delivered, with 4 sub-categories	Format	One-on-one visits	Conduct educational outreach visit
	Setting	In-person visits in clinical spaces	Conduct educational outreach visit
	Personnel	A detailer with background in public health, pharmacy, nursing, or related field	N/A
	Population	Detail staff involved in delivering or supporting the EBP.	Remind clinicians

Mapping each component to ERIC reveals that four AD common components pertain to conducting educational outreach visits, three to making training dynamic, and one each to seven other strategies: develop a formal implementation blueprint, tailor strategies, distribute educational materials, develop and implement tools for quality monitoring, train facilitator, provide ongoing consultation, and remind clinicians (Table 2). Taken together, Table 2 shows that, as commonly used, AD programs are a multi-faceted implementation strategy that bundle together a range of educational and interactive components.

### MIDAS AD program tailoring decisions

Key to beginning the program was identifying potential modifications of AD components, questions to consider for our program, and the MIDAS-specific context from which all decisions were made (Table 3). We strove to apply a consistent MIDAS AD strategy to all four EBP interventions. While based on our experiences developing the AD program, we structured this paper around providing guiding questions and points of consideration for future AD program developers when making tailoring decisions.

**Table 3:** Potential AD modifications based on FRAME-IS modules.

Module 2: “What”	Module 1: Description		Module 3: “Nature” of modification	Module 4: “Rationale”	
	AD Component	AD Subcomponent	Potential Modifications	Questions to Consider	MIDAS context with tailoring decision made
Content	Campaign Development	Use behavior change theories to inform outreach strategys	1. Use an individual theory of behavior change (e.g., Theory of Planned Behavior, Self-determination Theory) 2. Use a health-specific theory (e.g., Health Belief Model, Health Action Process Approach) 3. Use an organizational theory (e.g., Diffusion of Innovations, Normalization Process Theory) 4. Integrate multiple theories.	• What is the nature of the innovation (e.g., technological, psychosocial)? • What implementation stage is the setting at (e.g., pre-implementation, sustainment)? • What level is the change intended to affect (individuals, teams, organizations, etc.) • Based on answers above, what other theories might be more applicable?	VHA ADS already uses Theory of Planned Behavior, therefore MIDAS retained this as the underlying theory.
		Set goal of improving adherence to EBP	None, we considered this to be core.	N/A	N/A
		Develop key messages based on evidence for topic	None, we considered this to be core.	N/A	N/A
		Develop EBP materials (e.g., practitioner guide, quick reference guide, patient materials)	1. Use existing materials from other AD programs or EBP developers. 2. Adapt existing materials. 3. Develop only internal materials (e.g., detailer guidance) 4. Create written materials. 5. Create digitized materials.	• Are there existing materials? • Do providers use printed materials? • Do providers see patients in-person to deliver materials? • Will detailing be in person or virtual? • Would providers benefit from reference guide?	One program used existing materials (Clinician Brochure and Provider Guide)
		Conduct preliminary interviews with front-line staff^a^	1. Survey staff regarding needs. 2. Integrate key informants into AD team. 3. Omit.	• Are barriers already known? • Does the AD program have capacity to conduct and analyze interviews or surveys? • Are providers likely to participate in interview or surveys? • Are detailers already integrated into the setting?	The MIDAS team was not previously integrated into the setting, so we interviewed staff to better understand setting needs.
	Campaign Initiation and Recruitment	Create templates for recruitment communication (e.g., email scripts, call scripts)	1. Provide standards but allow detailers to tailor communication. 2. Omit.	• How experienced is the detailer? • Is it important to have standardized approach across detailers? • Are detailers willing to use or tailor templates?	We used templated emails with some personalization.

		Meet with clinical staff	None, we considered this to be core.	N/A	N/A
	In-session Delivery	Use NaRCAD approach (introduction, needs assessment, key messages, handling objections, summary, closing)	None, we considered this to be core.	N/A	N/A
		Deliver a presentation about research on the topic	1. Substitute presentation for conversational delivery of content. 2. Tailor the presentation based on preliminary interviews or other knowledge of the setting.	• Will the detailing be one-on-one or with a group? • Will technology be available? • Is new research about the EBP available that may not already be commonly known?	Following the NaRCAD model, the MIDAS team delivered a presentation on research on the EBP.
		Use motivational language to overcome barriers	None, we considered this to be core.	N/A	N/A
		Provide clinician with EBP materials (e.g., brochures, 1-pager, pocket cards)	1. Deliver digital materials. 2. Provide links to resources. 3. Suggest phone apps with salient information.	• Do clinicians prefer written materials or digital materials? • How can the materials be aligned with clinic workflows? • Do clinicians often see patients in one office, or move between offices?	The MIDAS detailer sessions were virtual, so we relied on digital materials with links or recommendations for other resources.
Evaluation	Collect outreach data (clinician demographics, method of outreach, length of visit, key messages covered, barriers, commitment to behavior change, recordings of visits)		1. Focus on data of most interest to program evaluation goals. 2. Omit data of less interest to program evaluation goals.	• Are detailer performance evaluations based on outreach activities? • What is a feasible amount of data for detailers to record? • Do have time to record visit information? • What are the primary goals of collecting outreach data?	The MIDAS team collected outreach data for evaluation purposes and for consistency with VHA ADS processes, using same variables as VHA ADS.
	Develop mixed methods evaluation structure^a^	Fidelity assessment^a^	1. Create a brief self-assessment. 2. Record outreach visits for detailer to self-observe then self-rate. 3. Omit.	• Are self-assessments accurate? • Is there a psychologically safe program culture for sharing self-criticism? • Are detailers allocated time to conduct fidelity assessments?	The MIDAS team used a comprehensive fidelity process to monitor consistency with our goals and identify areas for improvement.
		Peer Review^a^	1. Use a single reviewer. 2. Use two reviewers to triangulate perceptions. 3. Use a reviewer who is a trained detailer. 4. Use a reviewer who is a clinician targeted in the campaign. 5. Sample only a subset of outreach visits for review. 6. Incorporate independent/blinded peer review 7. Omit.	• Are trained peers available to conduct observations • Is there a psychologically safe program culture for sharing peer feedback? • Are deviations of fidelity, and standardization, important for program goals? • Do peers have sufficient knowledge and training of detailing and giving effective feedback?	The MIDAS team engaged two peer reviewers, one trained as a detailer, who watched recordings and provided feedback on a subset of the detailing visits. In later MIDAS projects, we incorporated independent peer review to compare and discuss differences in perceptions between the detailer and peer reviewer.
		Collect data on recipient satisfaction^a^	1. Deliver surveys to or interview clinicians who received detailing. 2. Request *ad hoc* feedback from informants within the setting conversationally or informally via email. 3. Omit.	• Do program staff have technical capacity to elicit and summarize clinician feedback? • Is clinician satisfaction part of program goals? • How burdened are clinicians in the setting? • Are survey response rates typically high in this setting?	The MIDAS AD program was new, thus we prioritized gathering participants’ feedback via survey and reviewed survey feedback regularly to discuss opportunities for improvement in our approach.
		Collect patient impact data^a^	1. Use electronic health record (EHR) review. 2. Solicit for clinicians to report on patient interactions and outcomes. 3. Survey or interview patients. 4. Omit.	• Do program staff have technical capacity to query and analyze EHR data, or conduct surveys or interviews? • Are patient survey response rates typically high in this setting? • Are there restrictions on surveying patients? • Is program funding dependent on demonstrating patient impact?	The MIDAS team included quantitative and qualitative data analysts. Thus, we were able to analyze patient outcomes from EHR data. Funders also required demonstrating evidence of patient impact.
Training	Train the detailer in basic AD skills.		1. Send detailer to an existing training (VHA ADS Basic Skills or NaRCAD 101) 2. Conduct an in-house training with experienced detailers. 3. Have new detailer apprentice with an experienced detailer.	• Are resources available to send detailer to a training? • Are experienced detailers available for apprenticeship or custom detailing?	MIDAS detailer completed both NaRCAD 101 and VHA ADS Basic Skills training because program had resources to do so, and these are considered best practice for AD training.
	Recurring meetings to discuss cases		None, we considered this to be core.	N/A	N/A
	Role plays/opportunities to practice		None, we considered this to be core.	N/A	N/A
	Subject Matter Expert Meetings^a^		1. Merge with role plays. 2. Merge with case discussions. 3. Omit.	• Is the detailer knowledgeable about the EBP? • Does detailer have time allocated to SME meetings? • Are there resources to attract SME’s or compensate their time? • Are SME’s willing to conduct role plays?	The MIDAS team worked with the developers of the EBP and conducted role plays with multiple VHA primary care providers and academic detailers who had experience detailing on inappropriate polypharmacy.
Context	Format	One-on-one visits	1. Conduct small group visits (i.e., huddles or team-level visits). 2. Conduct in-services for entire department or clinic.	• Are clinicians available for one-on-one visits? • Has site leadership supported clinicians’ participation in detailing? • Are clinicians more likely to attend group visits or in-services than individual sessions? • Are there scheduling challenges that might be solved by attending an existing meeting?	The MIDAS detailer met primarily one-on-one with clinicians. At the request of a local team, the MIDAS team conducted a single small group visit, then subsequently formalized a small group visit approach.
	Setting	In-person visits in clinical spaces	Conduct virtual visits.	• Are virtual visits common in the setting? • Are clinicians more likely to attend virtual or in-person meetings? • Is there concern that detailer may be seen as less credible if not physically in the setting? • Would virtual visits harm long-term relationship-building?	The MIDAS team originally planned to complete first AD visits in-person but switched to entirely virtual delivery due to the COVID-19 pandemic.
	Personnel	A detailer with background in public health, pharmacy, nursing, or related field	1. Hire a detailer with existing content expertise for campaign. 2. Hire a detailer based on what would seem most credible to clinicians. 3. Hire a detailer based on existing communication skills.	• Are detailers with appropriate expertise available? • What background would be most credible to clinicians? • Are available detailers skilled in interpersonal support and effective communication? • Do regulations or hiring restrictions impact staffing?	No single detailer would have expertise in all EBPs. Existing funding structures also precluded hiring of VHA clinicians.
		Engage site champions to spread the EBP^a^	1. Connect with local detailer(s) to assist in spreading the EBP. 2. Require leadership to identify and allocate time to site champions. 3. Omit.	• Can a champion be easily identified, e.g., via leadership or self-nomination or inside knowledge • Does leadership see it as a priority to have an internal champion for this EBP?	The MIDAS team identified site champions with support from local facility leadership as part of the recruitment process.
	Population	Detail staff involved in delivering or supporting the EBP	1. Detail only clinicians. 2. Detail only support staff. 3. Use information from preliminary interviews to identify which staff are most critical for EBP deliver.	• Which staff are most likely to deliver the EBP? • Which staff may play an integral role in sustaining the EBP?	MIDAS detailed staff that were most closely aligned with the EBP such as nurses, primary care providers, pharmacists, and mental health providers.

^a^ Component not common to AD. ERIC types supplied in Table 4.

### MIDAS AD tailored components classification

The list of tailored and additional MIDAS AD components is presented in Table 4 along with the reason for tailoring or adding. Out of eighteen common AD components, MIDAS AD program developers retained 12 components with no modification; these are therefore not included in Table 4 (but can be seen in Table 2). MIDAS tailored (i.e., substituted or refined) six of the common components. The MIDAS AD program developers then added an additional seven components by integrating strategies not usually included in common AD. Added components not otherwise found in common AD are denoted with an obelisk symbol. No components of common AD were removed from MIDAS AD.

**Table 4:** Tailored MIDAS AD components.

FRAME-IS Module 2: “What”	FRAME-IS Module 1: Description		FRAME-IS Module 3: “Nature” of modification	FRAME-IS Module 4: “Rationale”
	MIDAS AD Component	MIDAS AD Description		
	*ERIC type*			*Goal and Level*
Content	Use behavior change theories to inform outreach strategy.	Use Theory of Planned Behavior.	Refining a strategy.	Being consistent with AD format as usually delivered by VHA ADS, MIDAS team adopted Theory of Planned Behavior.
				*Increase appropriateness for practitioner.*
	Conduct preliminary interviews with front-line staff^a^	Conduct pre-implementation interviews with targeted sites to assess perceptions and needs, then use information to tailor key messages and communication strategies.	Integrating another strategy into the implementation strategy in primary use.	Desire to ensure fit of key messages required understanding staff perspectives and existing knowledge, also desired baseline understanding of barriers and facilitators to employing EBP.
	*Tailor strategies*			*Increase appropriateness for practitioner.*
Evaluation	Fidelity assessment^a^	Fidelity assessment developed to assess adherence to core MIDAS AD components (Supplementary Material B).	Integrating another strategy into the implementation strategy in primary use.	Desire for consistent strategy delivery across trials and settings. Also desired data for conducting process improvement.
	*Develop and implement tools for quality monitoring*			*Improve fidelity for implementer.*
	Peer Review^a^	Trained detailers to conduct peer review of AD sessions.	Integrating another strategy into the implementation strategy in primary use.	Desire for consistent strategy delivery across trials and settings. Also desired data for conducting process improvement.
	*Develop and implement tools for quality monitoring*			*Improve fidelity for implementer.*
	Collect data on recipient satisfaction^a^	Recipients of AD complete a post-visit satisfaction survey after each detailing session (Supplementary Material D).	Integrating another strategy into the implementation strategy in primary use.	Desired data for conducting process improvement and to assess provider perceptions of MIDAS detailer.
	*Obtain and use consumer feedback*	Detailer and implementation team use this to develop possible improvements.		*Increase acceptability for practitioner.*
	Collect patient impact data^a^	Monitoring patient prescriptions to assess practitioner behavior change after detailing.	Integrating another strategy into the implementation strategy in primary use.	Desire to understand downstream impact of AD, which could affect whether AD is sustained and scaled.
	*Develop and implement tools for quality monitoring*			*Improve sustainability for organization.*
Training	Train the detailer in basic AD skills.	Send detailer to existing trainings (VHA ADS Basic Skills and NaRCAD 101).	Refining a strategy.	Program had resources for both trainings, and these are considered best practice for training per VHA ADS.
				*Increase fidelity for implementer*
	Subject Matter Expert Meetings^a^	Detailer trained one-on-one and in group settings with subject matter experts to gain knowledge of each EBP.	Integrating another strategy into the implementation strategy in primary use.	Using AD for 4 different clinical areas meant that detailer required training on content to be perceived as credible to clinicians.
	*Use train-the-trainer strategies*			*Increase acceptability for implementer; Increase appropriateness for practitioner.*
Context	One-on-one visits	Conducted group sessions in addition to, and sometimes instead of, one-on-one visits.	Substitution of format.	Some settings did not have flexible clinic grids for scheduling detailing and required group sessions for efficiency.
	*Conduct educational outreach visit*			*Increase reach; Increase adoption; Increase acceptability for practitioner.*
	In-person visits in clinical spaces	Shifted to detailing virtually rather than in-person visits.	Substitution of setting.	COVID-19 pandemic shifted VHA rules regarding in-person interactions, then a new social norm was in place post-pandemic to hold meetings by video.
	*Conduct educational outreach visit*			*Increase reach; Increase adoption; Increase feasibility for organization.*
	A detailer with background in public health, pharmacy, nursing, or related field	Use of detailer without a VHA clinical role.	Substitution of personnel.	VHA compensation rules precluded using existing detailer or anyone with a VHA clinical role for study purposes.
	*N/A*			*Increase feasibility for implementer.*
	Recruit site champions to spread the EBP^a^	Recruit champions at each site to serve as internal detailers.	Integrating another strategy into the implementation strategy in primary use.	Desire for champions to spread EBPs beyond those who participated in detailing based on theoretical assumption that champions are critical for successful implementation.
	*Identify and prepare champions*			*Increase reach; Increase appropriateness; Improve sustainability for practitioner.*
	Detail staff involved in delivering or supporting the EBP	Detail staff most involved with target clinical issue and those with time to attend detailing session.	Refining a strategy.	Ensuring EBP uptake required educating staff most likely to use the EBP, which included nurses, primary care providers, pharmacists, and mental health providers.
	*Facilitate relay of clinical data to providers*			*Increase adoption for organization.*

Italics indicate the ERIC strategy name.

^a^ Component not common to AD.

In Table 4, the FRAME-IS codes show the category of “what” was modified (Module 2), provide a “description” of the modification (Module 1) with ERIC labels, and the “nature” (Module 3) and “rationale” (Module 4) of each modification. Two modules are not included in Table 4: Modules 6 and 7. The “when” (Module 5) was always during pre-implementation and planning was always planned/proactive, except for the use of group visits when the modification was during implementation and the planning was reactive. The “who” (Module 6) of decision-makers was always the MIDAS team.

Per Module 4 (“rationale”), reasons for modification primarily stemmed from needs at the provider and organizational levels, and in some cases the implementer level. The goals of each modification varied and aimed primarily to improve appropriateness, acceptability, adoption, and reach. Per ERIC type (shown in italics in the Module 1 column), the MIDAS AD program tailored components related primarily to the method of conducting educational outreach visits and developing and implementing tools for quality monitoring of AD.

Remaining results are organized according to the “nature” of modification (Module 3), first by those tailored (Module 1, no symbol in Table 4) and then by the integration of other strategies into AD (Module 1, denoted with obelisk symbol in Table 4). Underlined words indicate the categories of “what” (Module 2) was changed with italics to draw attention to specific codes.

#### Tailored MIDAS AD components

Tailored components refer to those common in AD but modified for MIDAS (Table 4). One common component of AD content was tailored by *refining the strategy* of using behavior change theories to inform outreach strategy. Among the available options, MIDAS used the Theory of Planned Behavior because it is consistently named by VHA ADS and NaRCAD as the key theory underlying provider behavior change after detailing. This uses detailer influence to affect provider attitudes towards the EBP, share data of what is expected in practice to affect subjective norms, listen to provider opinions on the EBP to foster a perception of control, and secure a provider's verbal intention to change practice to be EBP-consistent (35). One tailored component of AD training was similarly *refining the strategy* to educate the MIDAS detailer via VHA ADS's Basic Skills training and NaRCAD's AD 101 training. There are few options for AD training, and these are consistently used by VHA ADS detailers to enhance fidelity of implementing AD.

All common components of AD context were tailored to MIDAS. The *substitution of format* (offering both group and one-on-one sessions) and *substitution of setting* (only virtual sessions were practical) were made in response to the needs and restrictions of the setting. The format varied slightly across projects in reaction to a site's staff requests. One unplanned substitution of format occurred in the suicide prevention project when providers insisted it was infeasible to conduct one-on-one visits. This modification compromised the fidelity of AD, which is assumed to work best one-on-one. Nevertheless, in this project AD was delivered to a group of providers under the assumption that it could increase reach, adoption, and acceptability among staff with limited time. The *substitution of setting* deviated from the original MIDAS plan (28), shifting from in-person sessions to virtual. This reactive change was made during the pre-implementation stage in response to the COVID-19 pandemic and retained across all projects since video meetings had become a VHA norm. A *substitution of personnel*—employing someone who was not a pharmacist or nurse as detailer—was made for several reasons. Commonly, VHA ADS uses pharmacists whereas other AD programs use detailers with clinical service or public health backgrounds. Regulatory restrictions prohibited part-time hiring of both existing VHA detailers or providers, and financial limitations precluded hiring one directly onto the MIDAS staff. We believed with appropriate EBP upskilling, as described in the training section below, a detailer without clinical or public health experience could be an excellent detailer. The population modification simply *refined the definition* of the targeted audience (“front-line staff”). In practice VHA detailers often target primary care providers (5). In MIDAS, the decision was made to detail staff most involved with the target clinical issue. This varied by EBP and included nurses, pharmacists, primary care providers, and mental health professionals across clinical services. Detailing clinical staff with a salient role in delivering each EBP aimed to increase adoption under the assumption that potential EBP adopters would be those who would benefit most from detailing.

#### Integrating other strategies into MIDAS AD

Integrated components are those not found in common AD but added for MIDAS. When developing the MIDAS AD program, strategies not usually found in AD were integrated across all four “what” categories (labeled with obelisk marker in Table 4).

The modification to content was the addition of pre-implementation interviews with front-line staff at participating sites prior to each project launch. This change was made for two reasons: a desire to understand whether each campaign's key messages fit well with staff perspectives on the topic and to understand the baseline implementation barriers and facilitators. The overall goal of tailoring this aspect of campaign development was to increase perceived appropriateness of AD among the strategy recipients (VHA providers). Information gleaned from interviewees was used to optimize delivery of targeted key messages to ensure they were responsive to reported barriers and would resonate with recipients. For example, in the DOAC trial, the second of four, pre-implementation interviews highlighted a site-level variation in who was responsible (pharmacists or primary care providers) and how DOACs were managed (centralized vs. decentralized vs. a combination). With this information, we developed targeted messaging based on the identified management structure and responsible party.

MIDAS AD further integrated evaluation strategies not commonly found in AD programs to improve process fidelity and acceptability and to enable later evaluation of project success. We aimed to assess both the strategy itself and the quality of the detailer's delivery of the strategy. With recipient approval, the MIDAS detailer recorded outreach sessions with providers to allow for detailer reflection, evaluation, and quality assurance. The detailer completed a self-report fidelity assessment (36) after each session to assess adherence to AD components. Peer review of sessions was another strategy added; periodically, a subset of AD session recordings underwent peer review for fidelity ratings via an external observer. The peer reviewer shared notes with the detailer which were deliberated in weekly collaboration meetings. These meetings empowered the AD team to troubleshoot challenges and refine the detailing as needed. One project further tailored the peer review process to include blinded ratings by the peers, which were then compared to the detailer's self-assessment prior to collaboration meetings. This modification was made to strengthen the peer review process and better discern areas of protocol fidelity and deviation.

Collecting recipient satisfaction surveys after each session was another evaluation addition that served as a form of process feedback. Survey data was reflected upon as a group and used to improve detailing. Further, to assess impact of AD on service provision, MIDAS collected patient outcomes data and monitored referrals and patient prescriptions as a proxy of provider behavior change post-detailing. Integrating an outcome monitoring strategy served to enhance sustainability by creating a norm of data tracking to assess use of best practices.

A single additional strategy was integrated into detailer training: subject matter expert meetings. Because MIDAS applied AD to four different clinical areas, there was a need to train the detailer on content for each campaign to ensure the detailer was perceived by providers as a credible source of information. Notably, upskilling on campaign topics is common in VHA ADS detailing (23) but is not described broadly in the literature as a common AD component. The MIDAS detailer met with content experts both one-on-one and as a group. These meetings served to increase the detailer's topical expertise across disparate EBPs to accurately disseminate the EBP information. Increasing detailer's EBP-specific knowledge aimed to improve perceived appropriateness and acceptability of the detailer educating providers. These supplementary subject matter expert trainings were delivered across projects, including those where the MIDAS detailer had clinical experience (CBT and suicide prevention) under the assumption that expertise is enhanced through repeated exposure and feedback. Initial meetings were focused purely on acquiring the knowledge and learning communication norms about the intervention; later meetings included a role-playing component for the detailer. This was also an opportunity for the detailer to received targeted feedback from the subject matter expert.

One context addition was to recruit and train site champions. Providers were identified from each site to not only receive education, but also to encourage them to advocate for EBP use among colleagues. The intention behind this modification was to enhance EBP uptake by having champions promote use of the EBP internally, essentially ensuring spread beyond the reach of one-on-one detailing recipients. This proactive change aimed to increase reach, appropriateness, and sustainability by preparing an in-house advocate.

## Discussion

Developing effective implementation strategy modifications requires a complex synthesis of understanding local needs and which aspects of a strategy are core, which are useful, and which can be effectively modified. In this study, we defined the common components of AD and nested them within a conceptual framework from implementation science. We then outlined how and why we modified common AD components to develop and tailor the AD strategy to fit program-specific needs and context. We also illustrated a repeatable modification process using the FRAME-IS template. We hope this can be used as a model for how future AD programs can use existing variation as a strength, while still learning from prior work.

There are four important observations from the process of tailoring AD for MIDAS. First, few modifications were necessary, particularly in the content domain. AD is already a robust strategy with resources available from NaRCAD, VHA ADS, and more (5–7, 11, 12, 21, 23, 24). Second, the wide variation in AD programs may be because context choices, such as format, delivery, and detailer background, truly are modifiable (able to be changed without compromising strategy success) and not “core” (essential and indispensable) to AD. In fact, in our project, a feature considered core to AD by many programs (5, 7, 11, 24)—one-on-one outreach—was infeasible in some settings, but shifting to group educational sessions for one project effectively ensured that the key campaign messages were delivered to providers. Third, project-specific content education was practical, instead of hiring detailers with expertise in the content of each study (9, 10, 23). Our primary detailer had a background in counseling and was trained in motivational interviewing. Training modifications focusing on the specifics of the EBP, the AD key messages, and providers' likely individual barriers and facilitators to adoption made the MIDAS detailer knowledgeable across all four clinical topics. Finally, due to our program's research interests, we modified traditional programs by adding evaluation components that may be infeasible or unnecessary in some AD programs (12). Our tools could be helpful to other AD programs to assess detailer skill, detailer behavior, detailer-provider interaction, provider behavior, and patient outcomes. We felt the peer review process, in particular, enhanced AD rigor and fidelity to ensure consistent delivery across projects compared to self-assessment (36) or no evaluation at all. Collecting data on recipient satisfaction and provider prescribing behavior means that the effectiveness of AD can be empirically validated. Adding these components can build the AD evidence-base regarding where and how AD is effective.

### Implications for AD program development

Strategy tailoring is necessary to fit implementation processes to specific needs (3, 15, 37). Yet, changing an established strategy raises key questions: how much can it change? At what point has it changed so much that the critical elements are gone? Given the wide range of conceptualizations regarding AD key components and activities (5–8, 11, 12), our work to describe common AD components and their tailoring is an important step. One previous review that attempted to define AD characteristics found a 36.5%–100% variation between AD programs, particularly regarding communication components (12). A subsequent Delphi study (11) specified six key features of AD, among them an emphasis that AD focus on changing provider behavior. Despite these attempts at consensus, disagreement remains. For example, some argue AD is most effective in one-on-one, face-to-face sessions (6, 7, 9), while others report effective virtual delivery (10), or that—in contrast to the Delphi study findings—AD is designed for making workflow process improvements (21). This variation in what defines AD calls for future studies to document and make available programmatic components, resources used for development, and modifications from those resources. Our work starts to fill this aim.

While we propose that choices made for MIDAS *may* represent areas that are core (i.e., content) and modifiable (i.e., evaluation, context), we cannot conclusively say this is true across contexts. However, based on our literature reviews and experience across four projects, we can provide a template and guidance for tailoring AD programs. When we developed and delivered AD, there were many potential decision points for how to tailor the program. We describe these guiding questions to provide options for tailoring AD depending on context. Table 3 is a resource for AD developers to consider as they embark on their own implementation projects.

While several resources are freely available to learn about AD (10–12, 23, 24), we can only find one guidebook for creating and implementing a new AD program (25). To build the public library on AD development and study, MIDAS AD protocols and evaluation tools are available as supplementary materials. We strongly suggest that AD programs use the FRAME-IS template in Supplementary Material E to track their modifications towards building our understanding of which AD components are core and which are modifiable, in effort towards the broader study of implementation strategy bundles.

### Strengths and limitations

Strengths of this report are the use of extensive document review and coding schemes supported by a team of collaborators with strong implementation science experience, the application of a systematic method for reporting modifications, and the demonstration of a generalizable suite of modifications that applied to four projects with different EBPs. A limitation for non-VHA settings is that there may be more variations on common components than we identified, especially given the large number of small AD programs nationwide. Notably, in non-VHA settings the payment and reimbursement systems provide different prescribing incentives and adherence metrics. Another limitation is our inability to assess which modifications were effective for their programs.

## Conclusion

The present work is of greatest relevance to those seeking to develop and test new VHA AD programs and for implementation researchers interested in using FRAME-IS to identify critical and modifiable components of AD and other implementation strategies.

AD is a multi-component bundle of strategies; indeed, multi-faceted and tailored strategies are necessary to affect health providers' behavior (38). Implementation strategies are often bundled (3), which presents a challenge for studying their effects on outcomes (15). Efforts have been made to specify discrete strategies within bundles (13), track their effects on outcomes (3, 15), and document strategy modification (4). Still, few examples of combining these efforts to determine which modifications affect outcomes exist (39). The prominent barrier is that implementation scientists have yet to consistently take the prerequisite first step of unbundling strategies. The present work displays an effort to unbundle and define both the usual practice of AD, name the modifications present in a series of projects, and provide guidance for others seeking to tailor an AD program for their needs. Future analysis of the MIDAS projects will indicate the successes and failures of MIDAS AD and highlight AD components critical for implementation outcomes.

Our work to describe common AD represents an important step in studying AD given the wide range of conceptualizations regarding AD key components and activities (5–8, 11, 12). The FRAME-IS method used here may provide a template for other VHA AD programs to specify which components are core and modifiable across applications. We hope this contributes to future use and study of AD as an implementation strategy.

## Data Availability

The original contributions presented in the study are included in the article/Supplementary Material, further inquiries can be directed to the corresponding author.
