# Supplementary material for: Core and modifiable components of academic detailing: demonstration of implementation strategy development, tailoring, and documentation process
Source: Front Health Serv. 2025 Jun 3;5:1521504. doi: 10.3389/frhs.2025.1521504 (PMC12170586; doi:10.3389/frhs.2025.1521504)
Supplement: Supplementary file 1 [file Supplementaryfile1.docx]

**MIDAS Academic Detailing Training Guide**

**Purpose:** *to guide a new MIDAS academic detailer through activities associated with detailing before, during, and after the intervention*

**Table of Contents**

[Pre-intervention 3](#_Toc177989120)

[Establish project goals 3](#_Toc177989121)

[Site and Provider Identification 3](#_Toc177989122)

[Campaign Material Development 3](#_Toc177989123)

[Identify Site-level Barriers/Facilitators 4](#_Toc177989124)

[Identify Data Sources 4](#_Toc177989125)

[Prepare for outreach 4](#_Toc177989126)

[Baseline Survey Administration 5](#_Toc177989127)

[*May be administered before or as a part of initial outreach* 5](#_Toc177989128)

[Intervention 5](#_Toc177989129)

[Outreach 5](#_Toc177989130)

[Provider Visits 5](#_Toc177989131)

[Academic Detailing-related activities 6](#_Toc177989132)

[Peer review 6](#_Toc177989133)

[Post-intervention 7](#_Toc177989134)

[Champion check-ins 7](#_Toc177989135)

[18-month survey administration 7](#_Toc177989136)

[Academic Detailing Meetings 7](#_Toc177989137)

[AD Collaboration Meetings: 7](#_Toc177989138)

[AD Meetings 7](#_Toc177989139)

*Note: Activities are listed in the most likely chronological order, though many steps are inherently iterative and not always necessarily done in the order listed.

# **Pre-intervention**

*Includes startup activities that are ideally initiated at least* ***three*** *months before outreach*

### Establish project goals

Create project-specific timeline including significant timepoints

Determine target audience

Plan general outreach strategy

- A general rule of thumb is to reach out to providers a maximum of 3-4 times.
- You may decide to stage outreach based on capacity and number of eligible providers.

## Site and Provider Identification

Collaborate with project team to identify and engage participating sites

Develop site engagement strategies for each participating site

Participate in recruitment and kick-off calls with sites to describe detailing approach and strategies and answer questions

Once a site has been recruited, connect with project team to:

Identify eligible providers, those most likely to benefit from detailing. This may include:

- - Providers who deliver the evidence-based practice (EBP)
  - Providers who refer Veterans for the EBP

Load providers into internal tracking system

Identify a local champion

Plan site-specific outreach strategy

### Campaign Material Development

Identify or create Academic Detailing (AD) visit materials

Develop key messages

Draft the AD visit guide

Develop provider-facing guide or use existing materials from:

- VA National Academic Detailing Services (ADS)
- Academic literature
- Content experts

### Identify Site-level Barriers/Facilitators

Review pre-implementation data (from interviews, recruitment calls, etc. with participating sites) to:

Understand site-level models of care

Develop list of potential barriers and facilitators

Develop participant personas

### Identify Data Sources

Review project-specific data source(s) (e.g., dashboard)

### Prepare for outreach

Conduct role plays/practice visits

Internal role plays with team members and with external colleagues

An AD team member may provide a persona to role play along with necessary background information.

The Academic Detailer is blinded to the persona ahead of the role play, providing an opportunity to practice real-world scenarios.

Initial Champion Meeting

*Happens ideally* ***one month*** *prior to outreach*

Create a champion-facing presentation

Plan for champion meeting

Compile information from the pre-implementation interviews and recruitment/kick off calls to prepare the champion meeting template

Conduct Champion meeting

Reiterate project and meeting aims

Review the AD Champion role and engagement levels/expectations

Ask about the local model of care and any barriers/opportunities for improvement

Confirm eligible providers (or connect with someone who can confirm

Ask about outreach opportunities to engage providers (i.e., provider meetings)

Develop a plan/timeline for site participation

Document Champion meeting

## Baseline Survey Administration

## *May be administered before or as a part of initial outreach*

Prepare surveys using chosen platform

Notify/confirm planned survey release date with Champion

Send surveys in batch from survey platform

Inform Champion that baseline surveys were sent

# **Intervention**

## Outreach

Review overall project and site-specific outreach strategies

Draft/tailor outreach emails

Email/message providers

- Consider creating a Microsoft Bookings calendar to include in outreach emails
- Consider using instant messaging software like Microsoft Teams

Document scheduled visits and/or refusals

Connect with the Champion regularly to provide updates and brainstorm outreach strategies

Continuously review and update AD visit guide and other materials

## Provider Visits

**Prep for a visit** *(at least 30 min)*

Review project-specific AD visit guide

Review Champion meeting documentation

Review individual provider data (if available)

☐ Review provider-facing PowerPoint presentation

☐ Have survey link(s) prepared/handy prior to visit

☐ *If Visit 2:* Review Visit 1 notes

**During visit** *(generally 5-30 min)*

Arrive 2-5 minutes early to welcome provider

Follow flow of AD visit (refer to AD visit guide for details)

- Introduction
- Needs assessment
- Key messages/features/benefits
- Handling objections
- Summary
- Close

Share provider guide/provider-facing presentation/data as needed to support key message

*If a provider no shows for their visit:*

- Wait 5 minutes past visit start time to allow for late arrival
- After 5 minutes, send a message to ask if they will be joining the visit
- If no response after a few minutes, email the provider to reschedule the visit

**After visit** *(at least 60 min)*

Send provider a message/email with satisfaction survey link and any materials (i.e., provider guide)

Complete post-visit documentation

*If the session was recorded:*

Share recording with an AD team member

Request that team member review recording

Provide review deadline *(See Peer Review section)*

## Academic Detailing-related activities

Academic Detailing-related activities are those outside of one-on-one provider visits (i.e., presentation at departmental staff meeting). The preparation and associated tasks for an AD-related activity will vary greatly.

**After an AD-related activity:**

Share materials with those who attended

Complete post-activity documentation

## Peer review

The AD team aims to review at least 25% of recorded visits. Once you have completed your post-visit tasks (particularly, documentation), ask another detailer or team member to review your visit and provide feedback.

**The Peer Reviewer will:**

Watch/listen to recording

Fill out a reviewer version of the AD Outreach Visit Assessment Fidelity Tool

Compare ratings with the detailer’s version of the AD Outreach Visit Assessment Fidelity Tool for the same visit

Notify detailer that you have completed your review

Add any topics for discussion and/or differences in ratings to an upcoming AD Collaboration meeting agenda

# **Post-intervention**

## Champion check-ins

*Generally, happen* ***nine months*** *after initial outreach*

Schedule a 1/2-hour meeting with Champion

Plan for meeting with Champion

Review information from prior detailing visits and the initial Champion meeting

Conduct Champion check-in meeting

Document Champion check-in meeting

## 18-month survey administration

Plan survey administration timeline with team member(s) to avoid duplicate surveys and multiple asks at once (i.e., interview requests)

Prepare surveys in chosen platform

Send surveys in batch from platform

# Academic Detailing Meetings

## AD Collaboration Meetings:

*Designated meeting series for detailers to discuss visits and receive team feedback (peer review and fidelity)*

**Tasks associated with the AD collaboration meetings include:**

Scheduling/cancelling meetings

Setting and/or contributing to the meeting agenda

Reviewing AD visit recordings and providing feedback

Facilitating meeting, notetaking and circulating notes to team after the meeting

## AD Meetings

*Designated meeting series to discuss progress on current detailing efforts/tasks and plan for upcoming projects*

**Tasks associated with the AD meetings include:**

Scheduling/cancelling meetings

Setting and/or contributing to the meeting agenda

Collaborating with non-detailer team member(s) to present satisfaction survey data

Facilitating meeting, notetaking and circulating notes to team after the meeting
