# Supplementary material for: Core and modifiable components of academic detailing: demonstration of implementation strategy development, tailoring, and documentation process
Source: Front Health Serv. 2025 Jun 3;5:1521504. doi: 10.3389/frhs.2025.1521504 (PMC12170586; doi:10.3389/frhs.2025.1521504)
Supplement: Supplementary file 2 [file Supplementaryfile2.docx]

MIDAS Academic Detailing Visit Fidelity Tool

| 1. Record ID |  |
| --- | --- |
| 1. Is this form being filled out by the Academic Detailer or Peer Reviewer?   *(If Academic Detailer, please complete items 2a and 3-5 below. If Peer Reviewer, complete items 2b and 3a).* | Academic Detailer  Peer Reviewer |
| 2a*.* Detailer’s name |  |
| 2b. Reviewer’s name |  |
| 1. Detailing visit date |  |
| 3a. Date of peer review |  |
| 1. Today’s date |  |
| 1. Was session recorded? | Yes *(skip to #6)*  No *(list reason in 5a)* |
| 5a. *[If No was selected]* Why wasn’t the session recorded? | Participant declined  Technical issue  Other *(list)* |

| 6. Introduction (*Academic Detailer and Peer Reviewer complete items 6-13)* | NA | No | | Yes |
| --- | --- | --- | --- | --- |
| 6a. Introduced myself and/or summarized last visit and asked about experiences or progress made |  |  | |  |
| 6b. Clearly stated the reason/purpose for the visit |  |  | |  |
| 6c. Verified time available/allotted for the visit |  |  | |  |
| 7. Needs Assessment | NA | No | Somewhat | Yes |
| 7a. Used open-ended questions to gain an understanding of current practice/management strategies |  |  |  |  |
| 8. Key Message(s) Features & Benefits | NA | No | Somewhat | Yes |
| 8a. Discussed at least 1 key message but did not overwhelm the provider with too many key messages |  |  |  |  |
| 8b. Obtained commitment from provider to implement a change in behavior(s) |  |  |  |  |
| 9. Handling Objections | NA | No | Somewhat | Yes |
| 9a. Managed objection(s) to message(s) |  |  |  |  |
| 9b. Elicited and acknowledged barriers |  |  |  |  |
| 10. Summary | NA | No | Somewhat | Yes |
| 10a. Summarized key items discussed |  |  |  |  |
| 11. Closing Your Visit | NA | No | | Yes |
| 11a. Asked if there were additional questions/concerns |  |  | |  |
| 11b. Thanked the provider for their time |  |  | |  |
| 11c. Identified plan for follow-up (e.g., future visit) |  |  | |  |
| 12. Overall Review of Detail Visit | NA | No | | Yes |
| 12a. The conversation went smoothly |  |  | |  |
| 12b. The provider seemed engaged |  |  | |  |
| 12c. Overall, the encounter embodied the elements of a successful AD visit (e.g. Elicit, Provide, Feel, felt, found; reflections; resisted the “righting reflex”) |  |  | |  |
| 12d. It is feasible for the provider to implement the key points |  |  | |  |
| 12e. To the best of your judgment, this detailing session resulted in building/maintaining a positive relationship with the provider |  |  | |  |
| 13. During the session, how much did the Academic Detailer vs. the provider talk? | Detailer talked most of session  Detailer and provider talked equal amounts  Provider talked most of session | | | |
| **Academic Detailer Review (***Peer reviewer skip to item 17****)*** | | | | |
| 14. Areas for Improvement  (*You may use your "No" and/or "Somewhat" responses above to inform this section)* |  | | | |
| 15. Other comments |  | | | |
| 16. Are there specific topics you’d like to bring to an AD meeting? | Yes *(provide details below)*  No | | | |
| 16a. If yes, please provide details |  | | | |
| 16b. Please describe any resolution from the AD meeting |  | | | |
| **Peer Review** | | | | |
| 17. Peer Reviewer Comments |  | | | |
| 18. Note discrepancies between peer and detailer ratings |  | | | |
| 19. Are there specific topics you’d like to bring to an AD meeting? | Yes *(provide details below)*  No | | | |
| 19a. If yes, please provide details |  | | | |
| 19b. Please provide any resolution from the AD meeting |  | | | |

Adapted from: Smart M. H., Monteiro, A. L., Saffore, C. D., Ruseva, A., Lee, T. A., Fischer, M. A., & Pickard, A. S. (2020). Development of an Instrument to Assess the Perceived Effectiveness of Academic Detailing. *The Journal of Continuing Education in the Health Professions, 40*(4), 235–241. https://doi.org/10.1097/CEH.0000000000000305
