# Supplementary material for: Core and modifiable components of academic detailing: demonstration of implementation strategy development, tailoring, and documentation process
Source: Front Health Serv. 2025 Jun 3;5:1521504. doi: 10.3389/frhs.2025.1521504 (PMC12170586; doi:10.3389/frhs.2025.1521504)
Supplement: Supplementary file 3 [file Supplementaryfile3.docx]

**MIDAS VIONE**

Academic Detailing Visit Guide

**Contents**

[MIDAS VIONE Academic Detailing Goal 2](#_Toc177628063)

[Physician Visit Strategy 2](#_Toc177628064)

[Pharmacist Visit Strategy 2](#_Toc177628065)

[Academic Detailing Script 3](#_Toc177628066)

[Introduction: 3](#_Toc177628067)

[Needs Assessment: 4](#_Toc177628068)

[Key Message: 4](#_Toc177628069)

[Handling Objections: 7](#_Toc177628070)

[Summary: 7](#_Toc177628071)

[Closing: 7](#_Toc177628073)

[Appendix A: Barriers 8](#_Toc177628074)

[Responses to Physician Barriers 9](#_Toc177628075)

[Responses to Pharmacist Barriers 14](#_Toc177628076)

[Appendix C: Personas to help choose a key message 17](#_Toc177628077)

# MIDAS VIONE Academic Detailing Goal

Support clinicians in reducing inappropriate polypharmacy by deprescribing potentially inappropriate medications- those not vital or indicated in older adults using the VA-developed [VIONE](https://marketplace.va.gov/innovations/vione) framework and associated tools

## Physician Visit Strategy

| Detailers will focus on incorporating deprescribing/VIONE methodology into physicians’ daily workflow. The detailer may share dashboard data with the physician during the second visit. |
| --- |

**Provide one of the following key messages based on the conversation and physician’s needs:**

- During clinic visits, consider whether each medication falls into the category of V-I-O or N, then engage in shared decision making about stopping any potentially unnecessary medications.
  - *Optional: Invite them to use the CPRS VIONE discontinuation reasons for the VIONE program to track program implementation (since they will need to view the discontinuation screen anyway)*
- Place a polypharmacy consult to a pharmacist to assist with deprescribing, especially in challenging cases.
  - This differs by facility. The consult may be titled “drug information consult” or “PACT pharmacy window” or may involve including the pharmacist in an addendum to their notes.
- If they ask for more information about the dashboard and there is time, show the Potentially Inappropriate Medications (PIMs) Deprescribing Dashboard or the Primary Care VIONE Risk Dashboard (although not as useful during a 1:1 encounter).
  - If they ask, we can offer to send the report of their patients (use encrypted email and do not save on computer).

**For sites without pharmacists available to do polypharmacy consults or with substantially different clinic workflows, adaptation of these key messages may be required based on detailer judgment.*

## Pharmacist Visit Strategy

| Discuss the VIONE dashboards and help pharmacists navigate them. |
| --- |

*Before visit – Ask the Academic Detailing Champion to help determine if the pharmacist has a rough knowledge of VIONE and availability of tools.*

1. **Shared discussion about deprescribing using VIONE tools**

- What are they doing related to deprescribing? (workflow, are they using VIONE?)
- How are they using the tools?
  - What do they understand about the capabilities of the dashboards?
- Do they do deprescribing consults? If so, about how many? Maximum?
- Have they discussed the idea with their primary care patient-aligned care team (PACT)?
- What do they understand about the purpose of the VIONE risk score?

1. **Do they have a basic knowledge of VIONE tools?**

- If yes: Continue to step 3.
- If no: Share the PIMs Deprescribing Dashboard & Primary Care VIONE Risk Dashboard.
  - Orient them to the features of each dashboard.
- *Optional: Detailer can mention that the physicians and pharmacists can check the CPRS discontinuation reasons for the VIONE program to track program implementation.*

1. **Provide one of the following key messages based on the conversation and pharmacist’s needs:**

- Use the VIONE dashboard tools to identify patients for whom deprescribing may be indicated.
  - Focus on the high-risk patients (higher number of risk factors).
  - Review provider panel data with physicians to show them where there may be opportunities to reduce use of potentially inappropriate medications.
- Integrate VIONE into your daily workflow by incorporating the VIONE methodology into the visits they already have (for example, at the end of a diabetes visit).
  - *Optional: Fill out CPRS note/VIONE Polypharmacy Review template*
- Offer VIONE consults and send the physician a note with recommendations for them to consider.
- Use SharePoint resources for VA’s guidance on deprescribing certain drug classes and use the patient materials available on the VIONE PIMs Deprescribing dashboard for patients who have reluctance to deprescribe a drug.

# Academic Detailing Script

Introduction:

**Option A**: Good morning/afternoon. First, thank you for agreeing to meet with me today. I recognize that time is the one thing few of us have enough of. So, I understand we have about 15 minutes together; does that sound right? Ok, let’s dive in.

**Option B:** Good morning/afternoon. First, thank you for agreeing to meet with me today. Is now still a good time for a short chat about polypharmacy? Ok, great, I want to be respectful of your time, so let’s just jump in.

I’m [*name*], from the *[facility]* here today as part of a VISN-wide polypharmacy deprescribing initiative. This initiative is intended to get providers thinking more about deprescribing, especially in high-risk patients and to introduce a tool called VIONE that is being used to both simplify and encourage deprescribing.

A goal of this meeting is to talk about polypharmacy deprescribing, and more importantly to learn from your experience on the frontlines. I’m here as an ally, trying to figure out how we might work together to continue to improve patient safety.

Needs Assessment:

- Can you share with me your process for deprescribing medications for patients?
- What challenges have you encountered in addressing polypharmacy?
- When you hear “polypharmacy deprescribing,” what comes up for you?
  - Any particular challenges?
  - *If additional prompting is needed*:
    - One of the common messages from providers is that there isn’t a system in place for this.
    - When in a visit do you think about deprescribing?
    - How do you identify medications that are candidates to be deprescribed?
    - When there is a medicine that may be appropriate for deprescribing, how do you address it with patients?
    - Do you ever feel overwhelmed about a medication list or think the process of deprescribing would simply be too time-consuming in your brief visits?
      - *[Additional prompt if needed]:* How does that resonate with you?
- Can you tell me a bit about your approach to polypharmacy deprescribing?
  *[Employ Active Listening skills.]*
- *[Paraphrase what the provider just shared with you*.*]*
  - So, it sounds like it has been a challenge to… *[OR]*
  - What might help move the needle even more?
    - How familiar are you with VIONE- a new deprescribing approach that has been adopted by the VA? *[Explain briefly if not.]*
    - VIONE is a deprescribing methodology that focuses on deprescribing at opportune moments. It’s an easy way to quickly identify patients for whom deprescribing is indicated. *[Say more based on interest.]*
- I hear you; I can imagine how challenging that can be. *[Insert data relevant to noted challenge and/or benefit relevant to noted barrier.]*
  - *Example:* Polypharmacy is an opportunity to engage in shared decision-making with your patients.
- How familiar are you with VIONE?
  - VIONE has a few different components. Can you talk me through your understanding of what it is?
    - Are you aware of the dashboards? Have you ever used them? Are you comfortable using them?
    - Do you do VIONE consults? Would you be willing and able to?
    - Do you ever look at VIONE risk scores for patients or providers?

Key Message:

| **TAILOR YOUR KEY MESSAGE BASED ON NEEDS ASSESSMENT**  This should include the behavior you want to change. This is the pivotal point to address the provider’s specific challenge that they discussed during the Needs Assessment. Select a single key message; ask the provider to commit to taking immediate action and follow up with their results in 6 weeks. Ask what is feasible for them to do right now. |
| --- |

**General Key Message**

Would you consider taking a few minutes to explore with your patients whether medications are indicated then work to taper or discontinue those that aren’t? You may not be able to do this during every single visit, but maybe you can try this with your highest risk patients to help move the needle a bit.

- - - This is a team effort. I can work with your pharmacist to help quickly identify these patients or follow up later today with a list if you’d like to get started right away.
      - I’d like to hear how this is working or not working for you. How about we reconnect in about 6 weeks to see how it’s going?

| **Physician Key Messages** | **Pharmacist Key Messages** |
| --- | --- |
| During clinic visits, consider medications that fall into the category of O-N, then engage in shared decision making about stopping any potentially unnecessary medications. | The VIONE dashboard tools can help identify patients who may benefit from deprescribing.   - Focus on the high-risk patients (higher VIONE score). - Review provider panel data with physicians to show them where there may be opportunities to reduce use of potentially inappropriate medications. |
| Place a polypharmacy consult to a pharmacist to assist with deprescribing, especially in challenging cases.  Be aware that there is a risk score from 1-15 (with higher scores being riskier), and you have some patients with higher scores*.* | Integrate VIONE into your daily workflow by incorporating it into the visits you already have (for example, at the end of a diabetes visit).   - Run their full medication list . - Optional: Fill out CPRS note/VIONE Polypharmacy Review template. - Optional: Keep the dashboard on in the background for all visits, to look for patients with high VIONE risk scores or meds that could be deprescribed*.* |
| Before renewal of a medication, evaluate need for continuation. | Offer VIONE consults and send the physician a note with recommendations for them to consider. |
|  | Use SharePoint resources for VA’s guidance on deprescribing certain drug classes and use the patient materials available on the VIONE PIMs dashboard for patients who have reluctance to deprescribe a drug. |

**The Behavior Change “Ask”**

**Behavior Change:**

- In what ways would you be able to implement this into your practice?

*[Give the provider time to talk. Try to help them give specific answers about when they might use this.]*

**Asking for a Follow-up Visit:**

- Would you be willing to meet with me again in 6 weeks to share your results?

How often do you think you see patients that could benefit from a closer look at their medications?

*[Use their response to formulate a SMART goal. For example, if they see about 10 patients that could benefit, ask if they can try the (key message) you delivered on 3 patients in the next 6 weeks.]*

Handling Objections:

**The provider may have objections to the key message. Identify and address the barrier.**

*Discuss ways to overcome barriers and get their buy-in. Use Features (Facts) and Benefits [(What’s In It For me? (WIIFM)].*

- Yes, I see. That is a common concern we hear from providers. Would you like to hear about how they have addressed the situation? Would any of those work for you?
- You’re not alone in that thought. In what instances would you be willing to make a change with the medications?
- Can you share more about that?
- What might make it challenging for you to do this?
- What do you believe you need to feel comfortable about doing this?

*See chart in Appendix A for more information.*

Summary:

**Recap the visit and the provider’s commitment to the key message(s). Formulate this into a SMART goal and make the commitment clear to the provider so they know what will be discussed at the next visit.**

We agreed to a 15-minute visit, and I want to stay true to that, so let’s wrap up. We talked about some of the challenges with polypharmacy deprescribing, in your practice [*insert stated challenge*] seems to be a challenge. I introduced VIONE as a way to deprescribe when it’s opportune, focusing on those highest risk patients. You were generally agreeable to trying this out and letting me know how it was in a future visit. Anything important I’ve missed?

Are you able to pencil in our next visit now or shall I reconnect via email or Microsoft Teams? Great, thank you I’ll forward an invite or follow up via email or Microsoft Teams. Thank you so much for your time, if I haven’t run out of asks, I’m wondering if you’d be willing to a provide some feedback about our visit today. I’ll include the 2-question survey in my follow up email.

- At the next visit, would you like to see your VIONE dashboard data to see how your efforts are going and how many patients have been positively impacted?

Closing:

Do you have any other questions for me? Please feel free to reach out if you have additional questions. My contact information will be in the email, along with information you requested and the 2-question survey.

Thank you, again for your time today. I will see you in 6 weeks to see how things are going.

# Appendix A: Barriers

In general, it may be useful to respond to barriers with open ended questions before citing facts or providing recommendations. Please refer to the potential follow-up questions in the “Handling Barriers” section.

1. **The provider does not have time to utilize the VIONE tool.**
   1. Remind the provider that the deprescribing tool is already in their workflow in CPRS. They need to scroll down to select VIONE in the deprescribing drop down menu.
   2. Remind the provider that they can do a pharmacy consult.
2. **The provider does not know how to get started using the VIONE tool.**
   1. Offer support in training the provider how to utilize VIONE in CPRS.
   2. Have a quick reference guide to offer to the provider.
3. **The provider may not know which medications to start deprescribing.**
   1. Offer a list of medications to the provider that may be appropriate for deprescribing.
4. **The provider may not know which patients should be reviewed for deprescribing.**
   1. Encourage the provider to start with patients over 65 years old taking at least 5 medications or patients that are not adhering to medication.
5. **The provider is uncertain about risks/benefits.**
   1. 53,898 Veterans have been impacted by VIONE. 116,117 unique deprescribed medications and $4,124,507 in annualized cost avoidance. (Results from an assessment of outpatient data April 2016 to October 2019 indicate VIONE, launched in VISN 16, has had a measurable impact on Veteran care).
6. **The provider states some patients get their prescriptions from specialist and the provider does not feel comfortable deprescribing those medications or have time to work with specialists.**
   1. Ask if it is possible for the provider to set up a separate appointment to review medications or consult Pharmacy.
7. **The provider states that the patients may be fearful of going off medications that they have been taking for several years.**
   1. Offer the provider educational brochures that aid in starting a conversation with patients regarding deprescribing medications. Also let the provider know that they are the trusted touchpoint for patients and a frontline provider.
   2. It may be useful to frame the deprescribing as a “trial” to see how it goes.

## Responses to Physician Barriers

| **Objection** | **Follow-up questions/responses** |
| --- | --- |
| **General objections to deprescribing** | |
| Patients often don’t want their medications stopped.  Patients are unwilling to stop their meds. | You make a great point. How have you managed to address this challenge with patients in the past?  What do you think about framing the deprescribing as a “trial” to the patient, to see how it goes?  92% of older Medicare beneficiaries reporting being willing to stop 1 or more medication if told it was possible, and 67% wanted to reduce the number of medications. (doi:10.1001/jamainternmed.2018.4720.) |
| It’s challenging to have a patient stop [*drug X*]. | What do you find to be the biggest challenge in helping a patient stop [*drug X*]?  Encourage the provider to discuss with the pharmacist about how to stop specific drugs. Can note that deprescribing is a team effort and the pharmacist is there to help. |
| I don’t know how to convince patients to stop their medications. | Patients may be most receptive to hearing about possible side effects when convincing them to stop medications (doi:10.1001/jamanetworkopen.2021.2633). Encourage providers to try this approach with older patients, those with comorbidities or those taking many medications. Providers should start with medications that are not vital and important.  What do you think about framing the deprescribing as a “trial” to the patient, to see how it goes? |
| I don’t think this is a high priority for me to do. | 92% of older Medicare beneficiaries reporting being willing to stop 1 or more medication if told it was possible, and 67% wanted to reduce the number of medications. (doi:10.1001/jamainternmed.2018.4720.)  There is evidence that many of these drugs are also harmful. [Provide with Beers list.] I would be happy to get back to you with information about specific drugs you have questions about.  This is a priority for VISN 10, which sees this as a critical part of its journey to become a high reliability organization. Medications are such a common source of patient harm, that scrutinizing these drugs to prevent harm could really be worthwhile.  Taken individually that may be true. But, for VISN 10, leadership has made polypharmacy a priority because polypharmacy is a patient safety issue and considering all the centers within VISN 10 affects an increasing and significant portion of our population. Any little improvement from each site will add up and the overall impact will be on a significant portion of the VA population. |
| VIONE is the flavor of the month, i.e. VIONE is VISN 10 leadership quality initiative for the moment | How do you envision deprescribing will look in your clinic?  Acknowledge that VIONE may be the flavor of the month (VIONE is a tool among others to approach medication safety issues). However, the core concept of deprescribing is a fundamental part of medicine. Some doctors have found it useful to think about a “good prescribing continuum,” which beings with drug initiation, dose titration, switching or adding drugs, and stopping drugs.  Even if it is the flavor of the month, it could have a profound effect on patient safety. |
| Fear of adverse effects from stopping drugs | Share studies showing that deprescribing is generally safe.  https://www.uptodate.com/contents/deprescribing#H1706444822  Emphasize that deprescribing is a process, including re-evaluation of whether the deprescribing went OK. VIONE focuses on safe deprescribing, leaving the decision to the provider to identify patients who can safely be deprescribed. VIONE encourages providers to look for low-hanging fruits, start with a trial for example and monitor patient.  Consider a trial of deprescribing and monitor biomarkers of disease control when there is one (A1c, BP). |
| Some patients really need these drugs | Acknowledge that even Beers drugs may be appropriate in some patients. However, there’s lots of “low-hanging fruit.” Review the PIMs Deprescribing dashboard.  Acknowledge that the provider is right. Share that VIONE deprescribing approach focuses on safe deprescribing, leaving the decision to choose which patients and medications are appropriate for deprescribing to the providers. VIONE encourages the use of Beers list as a tool to identify lots of “low-hanging fruit.” that may not be obvious. Get provider opinion on using the Beers list to identify few appropriate patients. |
| Provider believes they are already doing the deprescribing, and they are asking about what more/or else to do | No one is doing perfectly, and the detailer may gently point out areas for improvement (e.g., “This is such a huge task, there are so many drugs, and polypharmacy is a complex. It sounds like you are doing the best you can. Are there any areas you are struggling with that I could help with?”)  Alternatively, the detailer may ask them how they do so well (feed into their confidence) to understand how they are doing it. (e.g., “Can you tell me more about how you are able to perform so well, it may be a good strategy to share with other sites.”)  The detailer may ask the provider, “What are areas you think others struggle with?” to find a barrier. Then, the detailer can explain how to handle that barrier. Can focus on the VIONE consult for providers. |
| Provider believes they are doing well but dashboard is showing otherwise. | Delicate issue- the detailer should tread carefully. Could speak to PIMs at site level but avoid focusing on provider performance (e.g., “At your site, we are looking at these PIMs being used”). Focus on “at your site” compared to their individual practice. |
| Provider who may not be interested in the second meeting because they believe they are already doing a great job. | Attempt to find the right barrier to focus on. If it is hard to find, the detailer may ask at the end of the visit if it would be helpful for the detailer to share the dashboard, maybe that could show them that there is room for improvement. Then, share their personal data in the next visit (if provider agrees to it as it is important to have permission to do so). |
| Provider is asking they were selected to participate | “Your clinic has made a commitment to having a greater impact.” Emphasize that we need all providers to play a role to make a difference. |
| Provider is already using VIONE | First approach:   - Encourage them to continue using VIONE. - Then, ask them how using VIONE is going. - See if anything can be improved upon.   Second approach:   - Ask if they would like the detailer to share their data from the dashboard. - See if anything can be improved upon. |
| **Key message 1: Before renewal of a medication during a clinic visit, evaluate the need for continuation** | |
| Many renewal requests come in by phone or portal. Then I renew drugs on the fly. Reviewing would require me to look back in the chart and do a lot of thinking, which takes a lot of time. Reviewing the need for the drug would also require me to call the patient and assess how they are doing much of the time. | For drugs on the Beers list, say that you will refill it now, but you’d like to revisit the ongoing need for the drug at the next visit, and make a note to yourself to do so. |
| **Key message 2: During clinic visits, consider whether each medication falls into the category of V-I-O or N, then engage in shared decision making about stopping any potentially unnecessary medications. [Talking point: A recommendation to stop carries a lot of weight.]** | |
| This takes a lot of time during already packed visits. | Try doing this only with the patients at high risk for polypharmacy complications. For example, patients over 65 years old on more than 10 drugs.  Consider having a visit dedicated only to deprescribing.  Cerner transition is coming. For each medication in CPRS, there will be steps in the future to associate it to a specific diagnosis. By getting rid of unnecessary drugs now, you can save time in the long run.  Consider switching to the key message 1 or 3. |
| I often don’t know all the drugs that a patient is taking. | Work with your R to specifically do a full medication reconciliation, including outside drugs, at the time of check-in. |
| I’m not sure which medications to stop. | Provide and summarize the Beers list.  Consider switching to key message 3 |
| What’s the evidence that VIONE is worthwhile? | VIONE is a new program that is still being evaluated. But the broader concept of deprescribing is gaining increasing recognition as an important part of the sound clinical care. |
| Lack of ownership of medications, not wanting to step on other providers’ toes (professional role and identity)  Uncertainty about why a patient is using a drug, or the intended duration (environment) | Consider switch to key message 3.  Ask your RN to coordinate communication with other providers around a specific medication. |
| How is this different than what I’m already doing? | Congratulate them on already doing deprescribing. Note that VIONE is a systematic approach to deprescribing to make sure none of the drugs fall through the cracks. |
| **Key message 3: Place a polypharmacy consult to a pharmacist to assist with deprescribing, especially in challenging cases.**  [Talking points: Need to cover what a polypharmacy consult is . The pharmacists have patient materials available on the VIONE PIMs Deprescribing dashboard for patients who have reluctance to deprescribe a drug. Important to note that they can help coordinate care among physicians, devise a taper, monitor symptoms afterwards.] | |
| My pharmacist has no time for this or does not offer this service. | VIONE is a VISN 10 priority. We’ve generally found that pharmacists want to serve the needs of physicians but don’t always know what their priorities are. If you discuss this at a PACT meeting, you might find that the pharmacist would be willing to help. |
| I don’t trust my pharmacist to do this. | One way that pharmacists can approach this is to review the medication list, then send their recommendations to you about which drugs may be unnecessary so you can have the final say. |
| I don’t know how to place a referral for polypharmacy management. | Demonstrate how to do it. [Note: Encourage them to submit a PACT Pharmacy Consult and write “VIONE visit” in the reason. Providers should know how to do this already, so it doesn’t require detailers to know the steps. However, the method of consulting Pharmacy may be different at each site, so worth discussing with AD Champion.]  Alternatively, the physician can add their pharmacist as a co-signer on a note where polypharmacy is addressed. |
| I am concerned that the patient will not come for an additional appointment. | Could ask a probing question like “How do you handle other referrals that require an appointment?” to elicit any strategies they use on handling the barrier with other referrals.  One potential solution could be a phone call/video visit vs. coming in-person for the appointment. |

## Responses to Pharmacist Barriers

| **Objection** | **Potential Responses / follow-up questions** |
| --- | --- |
| **General objections to deprescribing** | |
| Patients often don’t want their medications stopped.  Patients are unwilling to stop their medications. | 92% of older Medicare beneficiaries reporting being willing to stop 1 or more medication if told it was possible, and 67% wanted to reduce the number of medications. (doi:10.1001/jamainternmed.2018.4720.)  It may be useful to frame the deprescribing as a “trial” to see how it goes. |
| I don’t know how to convince patients to stop their medications. | Patients may be most receptive to hearing about possible side effects.  It may be useful to frame the deprescribing as a “trial” to see how it goes. |
| I don’t think this is a high priority for me to do.  Other problems have a high priority. | 92% of older Medicare beneficiaries reporting being willing to stop 1 or more medication if told it was possible, and 67% wanted to reduce the number of medications. (doi:10.1001/jamainternmed.2018.4720.)  There is evidence that many of these drugs are also harmful. [Provide with Beers list.] I would be happy to get back to you with information about specific drugs you have questions about.  This is a priority for VISN 10. |
| VIONE is the flavor of the month, i.e. VIONE is VISN 10 leadership quality initiative for the moment | How do you envision deprescribing will look in your clinic?  Acknowledge that VIONE may be the flavor of the month (VIONE is a tool among others to approach medication safety issues). However, the core concept of deprescribing is a fundamental part of medicine. Some pharmacists have found it useful to think about a “good prescribing continuum,” which beings with drug initiation, dose titration, switching or adding drugs, and stopping drugs.  Even if it is the flavor of the month, it could have a profound effect on patient safety. |
| **Key message 1: Use the VIONE dashboard tools to identify patients who may benefit from deprescribing. [Will need to review each of the different VIONE dashboards. Cover both the risk evaluation dashboard and the PIMS dashboard.]** | |
| I don’t know how to use the dashboard. | Show them how to use it. |
| **Key message 2: Use VA’s guidance for deprescribing strategies for certain drug classes (such as benzodiazepines, or PPIs), with the patient materials available on the VIONE dashboard for patients who have reluctance to deprescribe a drug.** | |
| This takes a lot of time during already packed visits. | Try doing this only with the patients at high risk for polypharmacy complications. For example, patients with VIONE risk scores of 4 or 5.  Consider having a visit dedicated only to deprescribing. You can get workload credit for VIONE visits. |
| I often don’t know all the drugs that a patient is taking. | Could you work with your support staff to do a full medication reconciliation, including outside drugs, at the time of check-in? |
| I’m not sure which medications to stop. | Provide and summarize the Beers list. |
| What’s the evidence that VIONE is worthwhile? | VIONE is a new program that is still being evaluated, but the broader concept of deprescribing is gaining increasing recognition as an important part of the sound clinical care. |
| **Key message 3: Integrate VIONE into your daily workflow. You can educate providers about the process for entering polypharmacy consults. You can also use VIONE template during encounters that are not specifically dedicated to polypharmacy.**  **For example, if you’re seeing a patient for a diabetes visit, or for hospital follow-up, try to fit a VIONE medication review in.** | |
| I have no time for this. | VIONE is a VISN 10 priority. We’ve generally found that pharmacists want to serve the needs of physicians but don’t always know what their priorities are.  Maybe talk about Cerner. Taking the time now, will save time post CPRS because there will be fewer meds. I think this i saying they don’t have time to do a consult. You might speak to the ease of making a consult and the benefit—frees you up to focus on patient care while knowing pharmacy is reviewing the meds. |
| I don’t have time set aside for doing VIONE activities.  This is not a type of consult pharmacists here offer. | Talk with your primary care providers and see if it would be possible to renegotiate the services that you are providing for them. Many physicians find this to be a very valuable service.  For patients at high risk for polypharmacy, squeeze in VIONE activities when you are seeing them for another reason. |
| **Key message 4: Review provider panel data with physicians to show them where there may be opportunities to reduce use of potentially inappropriate medications.** | |
| Providers don’t have time. | Discuss it at PACT meetings.  Add addendum to providers’ notes with the information you’ve learned.  Consider involving support staff to share the data from the dashboards with patients as they are seen in clinic. |
| Providers are not interested. | In our experience working with providers, we’ve found that most are quite receptive. Would you be willing to try it with 3 providers in the next month? |

# Appendix C: Personas to help choose a key message

| **Name** | **Main barrier** | **Key message** |
| --- | --- | --- |
| **“The overextended objector”**   - Frustrated with number of clinical demands - Deprescribing is the “flavor of the month” - Unaware of resources - Vaguely hostile | Low intention to address polypharmacy | Put in a consult to polypharmacy |
| **“The overwhelmed empathizer”**   - Would love to help - Uncertain when/how to address this without falling behind on workload | Knowledge of deprescribing practices | Put in a consult to polypharmacy |
| **“The knowledge needer”**   - Generally sympathetic to need for deprescribing - Flustered/overwhelmed with the challenge of the task - “This is just so complicated,” “How can I stop a drug that I didn’t start” - They’re on so many drugs - The medication list is never up to date | Logistical challenges to deprescribing | Put in a consult to polypharmacy |
| **“The deprescribing opportunist”**   - Sometimes does deprescribing if a medicine jumps out at her - Lacking a systematic approach | Maintaining/sustaining behavior change | Put in a consult to polypharmacy |
| **“The enthusiast”**   - “I love deprescribing” | Minimal barriers |  |
| **“The loner”**   - Would like to address polypharmacy, but does not feel supported by others on PACT team, including possibly the pharmacist | Support of staff | Sort meds by V-I-O-N |
