# Supplementary material for: Core and modifiable components of academic detailing: demonstration of implementation strategy development, tailoring, and documentation process
Source: Front Health Serv. 2025 Jun 3;5:1521504. doi: 10.3389/frhs.2025.1521504 (PMC12170586; doi:10.3389/frhs.2025.1521504)
Supplement: Supplementary file 4 [file Supplementaryfile4.docx]

**Academic Detailing Participant Survey**

This survey asks about your overall satisfaction with your Academic Detailing session (s)

- Years of practice: ___________________________
- Please indicate your title/role: ___________________________

**Post Visit 1**

| Please mark the box indicating your response below for each of the following questions after your **first** academic detailing session | | | | | |
| --- | --- | --- | --- | --- | --- |
|  | Not at all | Slightly | Moderately | Very | Extremely |
| The detailer was knowledgeable |  |  |  |  |  |
| The detailer was an effective communicator |  |  |  |  |  |
| This session was an effective way to get updated on EBP |  |  |  |  |  |
| I would be receptive to future visits |  |  |  |  |  |
| I would be receptive to future visits |  |  |  |  |  |
| The message I received about VIONE is feasible to implement in my practice |  |  |  |  |  |
| My practice is likely to change because of this visit |  |  |  |  |  |
| Please include any comments that can make our detailing better | | | | | |

**Post Visit 2**

| Please mark the box indicating your response below for each of the following questions after your **second** academic detailing session | | | | | |
| --- | --- | --- | --- | --- | --- |
|  | Not at all | Slightly | Moderately | Very | Extremely |
| The detailer was an effective communicator |  |  |  |  |  |
| The message I received about the VIONE is feasible to implement in my practice |  |  |  |  |  |
| Please include any comments that can make our detailing better | | | | | |

Adapted from Smart, M. H., Monteiro, A. L., Saffore, C. D., Ruseva, A., Lee, T. A., Fischer, M. A., & Pickard, A. S. (2020). Development of an Instrument to Assess the Perceived Effectiveness of Academic Detailing. *The Journal of Continuing Education in the Health Professions, 40*(4), 235– 241. https://doi.org/10.1097/CEH.0000000000000305.
